# Identifying the key genes and pathways in the progression of hepatitis C virus induced hepatocellular carcinoma using a systems biology approach

**DOI:** 10.1186/1471-2105-12-S7-A4

**Published:** 2011-08-05

**Authors:** Siyuan Zheng, Zhongming Zhao

**Affiliations:** 1 Department of Biomedical Informatics, Vanderbilt University Medical Center, Nashville, TN, 37232, USA; 2 Department of Cancer Biology, Vanderbilt University Medical Center, Nashville, TN, 37232, USA

## Background

Incidence of hepatitis C virus (HCV) induced hepatocellular carcinoma (HCC) has been increasing in many developed countries including the United States and Europe during the recent years. Although many efforts have been made to understand the pathogenesis, the picture of its progression still remains elusive.

## Materials and methods

We developed a systematic approach to identify deregulated biological networks in HCC by integrating gene expression profiles [1] with high-throughput protein-protein interaction data [2]. Samples were grouped into five disease stages including normal, cirrhotic, dysplastic, early and advanced HCC. For each pair of consecutive stages, we compared gene expressions and then mapped these measures to the protein interaction network. Responsive subnetworks were then identified from these node weighted networks. The searching algorithm is adapted from a previous study [3], which expands the seed graphs under constrains of several parameters.

## Results

Four networks were identified including precancerous networks (normal-cirrhosis and cirrhosis-dysplasia) and cancerous networks (dysplasia-early HCC, early-advanced HCC). A summary of these networks is shown in Table 1. An independent dataset was used for network validation. Statistical significance of these networks was assessed within three hypotheses. Little overlap was observed between precancerous and cancerous networks, in contrast to a substantial overlap within precancerous or cancerous networks. Network functions were annotated with Gene Ontology biological process using hypergeometric distribution based enrichment analysis. Significant functions were then assembled into a module map in temporal order. The apoptosis gene *ZBTB16* was highlighted by examining the module map, which shows a negative expression pattern with *c-myc*. Network analysis led to the identifications of key genes and pathways by developmental stage, such as *LCK* signaling pathways in cirrhosis, *MMP* genes and *TIMP* genes in dysplastic liver, and *CDC2*-mediated cell cycle signaling in early and advanced HCC. *CDC2*, a cell cycle regulatory gene, is particularly interesting because it is a hub protein of the module that shows correlative pattern with cancer progression.

**Table 1:** Overview of the responsive networks.

Network	#Genes	#Interactions	#DEGs^*^	#Hub interactions^†^
Normal- cirrhosis	55	67	53 (96.3%)	42 (62.7%)
Cirrhosis- dysplasia	38	50	37 (97.4%)	35 (70.0%)
Dysplasia -early HCC	60	65	53 (88.3%)	37 (56.9%)
Early- advanced HCC	68	98	59 (86.8%)	79 (80.6%)

^*^Differentially expressed genes (DEGs) were identified as genes with up or down regulation fold change ≥ 2 and student t test *P* value ≤ 0.01.

^†^Hub interaction number refers to the total number of interactions involving hub genes.

^‡^Hub genes were defined to have at least 5 interactions in each network.

## Conclusions

Our study uncovers a temporal spectrum of functional deregulation and prioritizes key genes and pathways in the progression of HCV induced HCC. Despite the confirmation of much knowledge in the pathogenesis of this disease, these findings also provide additional insights for further investigations.
